# The Relationship between Sleep Bruxism and Obstructive Sleep Apnea Based on Polysomnographic Findings

**DOI:** 10.3390/jcm8101653

**Published:** 2019-10-11

**Authors:** Helena Martynowicz, Pawel Gac, Anna Brzecka, Rafal Poreba, Anna Wojakowska, Grzegorz Mazur, Joanna Smardz, Mieszko Wieckiewicz

**Affiliations:** 1 Department and Clinic of Internal Medicine, Occupational Diseases, Hypertension and Clinical Oncology, Wroclaw Medical University, 50-556 Wroclaw, Poland; helenamar@poczta.onet.pl (H.M.); sogood@poczta.onet.pl (R.P.); ania.wojakowska@wp.pl (A.W.); grzegorzmaz@yahoo.com (G.M.); 2 Department of Hygiene, Wroclaw Medical University, 50-345 Wroclaw, Poland; pawelgac@interia.pl; 3 Department and Clinic of Pulmonology and Lung Cancer, Wroclaw Medical University, 53-439 Wroclaw, Poland; anna.brzecka@umed.wroc.pl; 4 Department of Experimental Dentistry, Wroclaw Medical University, 50-425 Wroclaw, Poland; joannasmardz1@gmail.com

**Keywords:** sleep bruxism, obstructive sleep apnea, polysomnography, diabetes

## Abstract

Obstructive sleep apnea (OSA) is the most common sleep disorder. Sleep bruxism (SB) is a masticatory muscle activity during sleep that commonly co-occurs with OSA. The presented study aimed to assess this relationship and to identify factors affecting this co-occurrence. Adult patients (*n* = 110) were evaluated for OSA and SB in a sleep laboratory using polysomnography. The episodes of bruxism and respiratory events were scored according to the standards of the American Academy of Sleep Medicine. The prevalence of OSA and SB was found to be 86.37% and 50%, respectively. The bruxism episode index (BEI) was increased in the group with mild and moderate OSA (apnea–hypopnea index (AHI) <30) compared to that in the group with severe OSA (AHI ≥ 30) (5.50 ± 4.58 vs. 1.62 ± 1.28, *p* < 0.05). A positive correlation between AHI and BEI was observed in the group with AHI < 30. Regression analysis indicated that higher AHI, male gender, and diabetes were independent predictors for the increased BEI in group with AHI < 30. The relationship between OSA and SB depends on the degree of severity of OSA. OSA is correlated with SB in mild and moderate cases of OSA in the group of patients with increased risk of OSA.

## 1. Introduction

Obstructive sleep apnea (OSA) is a common sleep disorder characterized by a collapse of the upper airways in the setting of continued respiratory effort, leading to airflow cessation and arterial oxygen desaturation, often terminated by arousal. OSA has been independently associated with cardiovascular diseases such as hypertension [1], stroke [2], myocardial ischemia [3], and arrhythmias [4] and mortality [5]. A relationship between OSA and sleep bruxism (SB) has been previously demonstrated [6]. The problem seems to be significant due to the high prevalence of OSA and SB. The prevalence of OSA ranges from 9% to 38% [7], and the prevalence of SB is estimated to occur in 13% of adults [8]. Thus, OSA may be one of the most frequent risk factors for SB in the adult population. SB has been defined as a masticatory muscle activity during sleep that is characterized as rhythmic (phasic) or nonrhythmic (tonic). According to the American Academy of Sleep Medicine (AASM), bruxism is a repetitive jaw-muscle activity characterized by clenching or grinding of the teeth and/or by bracing or thrusting of the mandible [9]. The International Classification of Sleep Disorders (ICDS-3) indicates the following clinical criteria for the classification of sleep bruxism: (A) the presence of regular or frequent tooth grinding sounds occurring during sleep and (B) the presence of one or more of the following clinical signs: (1) abnormal tooth wear consistent with the above reports of tooth grinding during sleep and (2) transient morning jaw-muscle pain or fatigue; and/or temporal headache; and/or jaw locking upon awakening consistent with the above reports of tooth grinding during sleep [10]. The most recent hypotheses on the etiology of SB support the roles of the central and autonomic nervous systems in the genesis of SB [11]. Most SB episodes occur during cortical arousal associated with an increase in heart rate [12]. Emotional stress; certain groups of drugs; consumption of tobacco, alcohol, or coffee; OSA; and anxiety disorders are recognized as important risk factors of bruxism among adults [13]. Recently, the relationship between SB and OSA has received much attention [14]. OSA has been considered as a new risk factor for SB [15]. However, the data on the association between SB and OSA are contradictory. Although the association between SB and OSA has been discussed in earlier studies [14,16], these studies have failed to confirm this relationship [15,17].

Therefore, in the present study, we aimed to assess the relationship between SB and OSA and to identify factors affecting this relationship.

## 2. Material and Methods

In this study, 110 adult patients were enrolled between March 2017 and March 2019. All subjects were suspected to have OSA and were hospitalized in the Department and Clinic of Internal Diseases, Occupational Diseases, Hypertension, and Clinical Oncology at the Wroclaw Medical University.

Inclusion criteria were as follows: age between 18 and 90 years, clinical suspicion of OSA, and willingness to participate in this study. Exclusion criteria were as follows: presence of neurological disorders and/or neuropathic pain, respiratory insufficiency, active inflammation, treatment with or addiction to analgesic drugs and/or drugs that affect muscle and breath function, presence of active malignancy and severe mental disorders, and cognitive disability.

All patients underwent overnight diagnostic polysomnography using Nox-A1 (Nox Medical, Reykjavik, Iceland) in the Sleep Laboratory of the Department and Clinic of Internal Medicine, Occupational Diseases, Hypertension, and Clinical Oncology at the Wroclaw Medical University, Poland. Polysomnograms were assessed in 30 s epochs in accordance with the AASM standard criteria for sleep scoring. Polysomnography (PSG) outcome variables included sleep latency, total sleep time (TST); sleep efficiency (%); and the percentages of N1, N2, N3, and rapid eye movement (REM) sleep. Abnormal respiratory events were scored from the pressure airflow signal evaluated in accordance with the standard criteria of the AASM Task Force [18]. Apneas were defined as the absence of airflow for ≥10 s. Hypopnea was defined as a reduction in the amplitude of breathing by ≥30% for ≥10 s with a ≥3% decline in blood oxygen saturation or arousal. The arterial oxygen saturation (SpO_2_) was measured with finger pulse oximetry.

SB was assessed by bilateral masseter electromyography (EMG), and the audio and video evaluation bruxism episodes were scored according to the standards of the AASM in three forms: phasic, tonic, and mixed. For the consideration of SB, EMG bursts should not be separated by >3 s to be considered part of the same episode, and EMG activity had to be at least twice the amplitude of the background EMG [19]. The scoring and manual analysis of the collected data were performed by a qualified physician (HM) from the Sleep Laboratory of the Wroclaw Medical University, Poland.

Statistical analysis was performed using the “Dell Statistica 13” software (Dell Inc., Round Rock, TX, USA). Quantitative data are presented as mean and standard deviation. Qualitative variables are expressed as percentage values. Significant statistical differences between arithmetic means were determined by the Mann–Whitney U test and between percentage values by the chi-square test. To determine the relationship between the analyzed variables, a correlation and regression analysis was performed. Parameters of the model obtained in the regression analysis were estimated using the least squares method. Moreover, the test accuracy was assessed based on receiver operating characteristic (ROC) analysis. Statistical significance was set at *p* < 0.05.

This study was approved by the Ethical Committee of the Wroclaw Medical University (ID KB-195/2017) and was conducted in accordance with the Declaration of Helsinki. All patients signed an informed consent form for participating in this study. Clinical Trial Registration: www.ClinicalTrials.gov, identifier NCT03083405.

## 3. Results

The mean age of all participants was 51.02 ± 14.19 years. Women constituted 40% (*n* = 44) of all the participants. The mean BMI was 28.93 ± 5.52 kg/m2. Diabetes and ischemic heart disease were diagnosed in 11% (*n* = 12) and 7.27% (*n* = 8) of the study patients, respectively. Hypertension was diagnosed in 45% (*n* = 50) patients.

The mean AHI and mean BEI were 23.28 ± 19.98 and 3.70 ± 4.27, respectively. The polysomnographic parameters in the studied group are presented in Table 1.

The prevalence of OSA (AHI ≥ 5) was 86.37% (*n* = 85) in the studied group. SB (BEI ≥ 2) was diagnosed in 50% (*n* = 55) of the studied patients. The prevalence of mild, moderate, and severe OSA and of mild/moderate and severe SB is presented in Table 2.

SB (BEI ≥ 2) occurred significantly more frequently in the group with OSA (AHI ≥ 5) than in the group without OSA (AHI < 5) (53.7% vs. 26.7%, *p* < 0.05). The incidence of SB in groups with mild, moderate, and severe OSA was: 61.6%, 64.3% and 35.3%.

**Figure 1:**
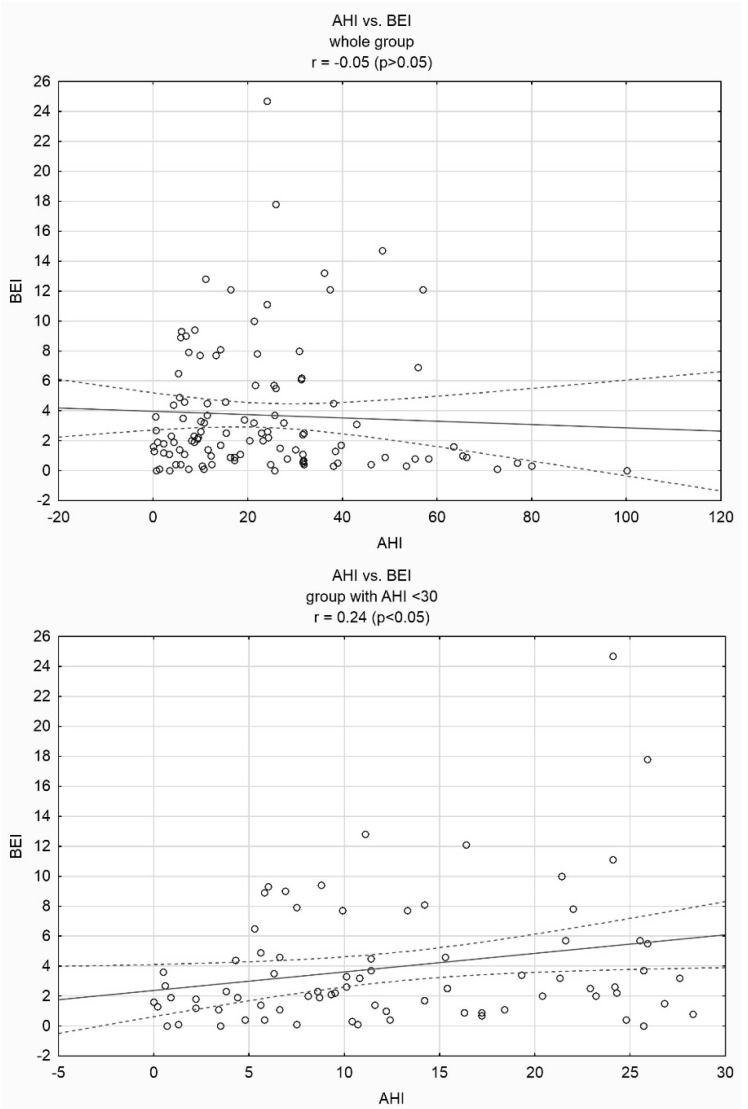

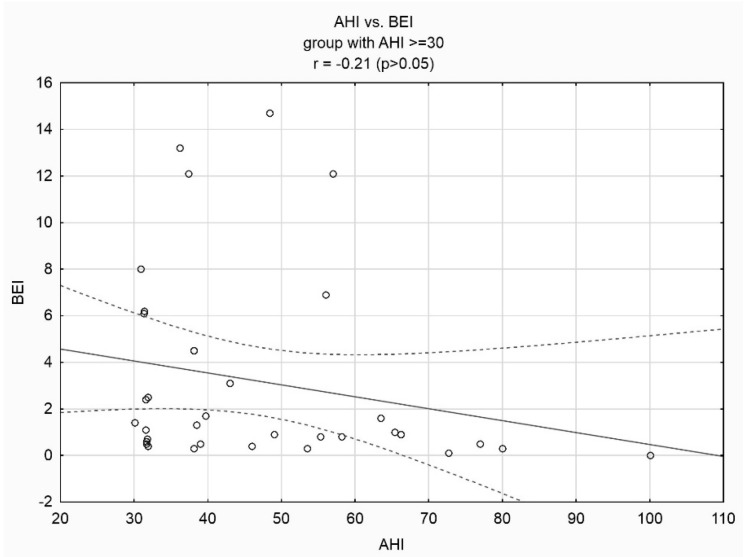
Correlation scatter plots between apnea–hypopnea index (AHI) and bruxism episode index (BEI) in the whole group, in the group with AHI < 30 and in the group with AHI ≥ 30.

No statistically significant correlation was found between AHI and BEI in the entire group (r = −0.05, *p* > 0.05, Figure 1). A positive linear correlation was observed between BEI and arousal index (r = 0.21, *p* < 0.05) and between phasic bruxism and arousal index (r = 029, *p* < 0.05) in the entire group. We also found a positive linear correlation between AHI and BEI (r = 0.24, *p* < 0.05, Figure 1) and between AHI and phasic bruxism (r = 0.27, *p* < 0.05) in the group with mild and moderate OSA (AHI < 30). No such correlations were observed in the group with severe OSA (AHI ≥ 30; r = −0.21, *p* > 0.05, Figure 1). Furthermore, no correlations were observed between AHI and tonic or mixed bruxism in both studied groups. In the group with AHI < 30, BEI was increased compared to that in the group with AHI ≥ 30 (5.50 ± 4.58 vs. 1.62 ± 1.28, *p* < 0.05). BEI was also increased in the group with OSA (AHI ≥ 5) compared to that in healthy subjects (AHI < 5) (4.03 ± 4.48 vs. 1.62 ± 1.28).

A positive linear correlation was observed between phasic bruxism and oxygen desaturation index (ODI) and between phasic bruxism and minimal oxygen saturation in the group with AHI < 30 (Table 3).

BEI was increased in patients with diabetes compared to that in patients without diabetes (5.78 ± 5.27 vs. 2.59 ± 4.17, *p* < 0.05).

**Figure 2:**
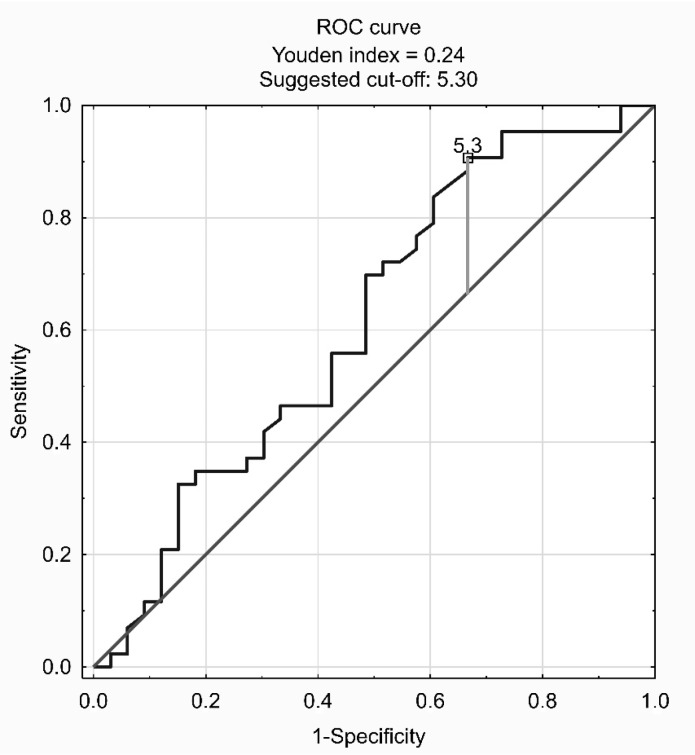
Receiver operating characteristic curve (ROC) suggesting the optimal apnea–hypopnea index (AHI) cutoff point for indicating its suitability to recognize bruxism (BEI ≥ 2) in the group with AHI < 30.

The cut-off point for AHI to predict bruxism (BEI ≥ 2) in the group with AHI < 30 was determined on the basis of the ROC curve (Figure 2). According to the ROC curve, the cutoff point was set at AHI = 5.3. In this group, the criterion AHI > 5.3 indicates bruxism with sensitivity and specificity of 0.533 and 0.907, respectively, which gives a prediction accuracy of 0.658.

Subsequently, to determine the factors that were independently associated with BEI in the studied patients, a regression analysis was made. In the group with AHI < 30, using a multivariate regression analysis and considering all potentially independent variables that were statistically significant in univariate models (male gender, age, diabetes, coronary artery diseases, AHI and arousal index), model 1 was obtained. Then, stepwise (in each step by removing the variable with the highest *p* value from the model), models 2–4 were obtained (Table 4). The highest determination coefficient R^2^ (0.488) was shown for model 1: BEI = 0.92 + 0.12 AHI + 1.73 male gender + 1.59 diabetes ± 3.82. On the basis of the obtained model, it can be stated that higher AHI, male gender, and diabetes were independent predictors for increased BEI in the studied patients.

## 4. Discussion

The most important result of this study is the positive correlation between BEI and AHI in the group with mild and moderate OSA. This result indicates the effect of the severity of OSA on the occurrence of correlation between AHI and BEI. The correlation was also observed in the group with AHI < 30. However, no such correlation was observed in patients with a severe form of OSA (AHI ≥ 30). One of the hypotheses linking SB and OSA is that SB activity protects against OSA by protruding the mandible and restoring airway patency [20,21]. However, this mechanism may not be adequate to prevent the airway from collapsing in severe OSA. In severe OSA, more effective mechanisms may be involved, e.g., excessive respiratory effort and/or increased respiratory rate, leading to a reduction in bruxism episodes. Thus, the probable explanation of this phenomenon is the limited role of bruxism as a protective factor in severe OSA. In this study, we did not investigate other protective mechanisms against OSA events.

The results of our study may explain the contradicting results of studies that investigated the correlation between OSA and SB. This correlation may depend on the studied population. The correlation may be observed if mild or moderate OSA is predominant in the studied population. However, if severe OSA prevails in the studied population, then the attempt to find a correlation may fail. It is worth noting that in many studies on the association between SB and OSA, insufficient research methods have been used, e.g., a telephone survey was used to diagnose SB [22], or self-administered questionnaire and clinical examination were conducted [23]. Studies that used polysomnography to diagnose SB in the studied groups are few [16,17,24,25]; hence, the results of these studies should be interpreted with caution. Recently, a correlation between OSA and bruxism was found in polysomnographic studies [26].

In the present study, we demonstrated that OSA (AHI ≥ 5), male gender, and diabetes are the independent risk factors for increased BEI. Numerous risk factors for SB in the general population have been reported. Caffeine, smoking, stress, alcohol, and anxiety are well-known risk factors for SB [13,22]. Reflux esophagitis [27], depression [28], and nocturnal frontal lobe epilepsy [29] were also described as a risk factor for bruxism. Few studies indicate OSA as a risk factor for bruxism [14,26,30]. Thus, the results of our study are in agreement with the findings of these studies.

In the present study, we found diabetes as a new potential risk factor for bruxism. Data on the association between diabetes and bruxism are very limited. Diabetes is associated with cardiovascular neuropathy, which results in a decrease in parasympathetic tone and sympathetic overactivity, similar to that occurring in bruxism [31]. Bruxism is considered as a protective factor in impaired salivation. Decreased salivary flow also occurs in diabetes [32]; thus, these findings may explain the increased risk for SB in diabetes.

Transient hypoxia commonly occurs in OSA, which is considered as a risk factor for bruxism. Moreover, hypoxia was previously described as a factor potentially associated with the onset of bruxism episodes [26,33]. The present study also showed a positive correlation between phasic bruxism and minimal SatO_2_ and between phasic bruxism and ODI; these findings confirm the association between hypoxia and SB.

It is worth noting that the prevalence of bruxism was quite high in the studied group with clinician suspicion of sleep apnea. SB was diagnosed in 50% of the studied subjects. A recent study by Tan et al. showed an SB prevalence of 33% in patients with OSA. Interestingly, the prevalence of SB is estimated at 12% in the general population [8,34] thus, the prevalence of SB in patients with OSA is higher than that in the general population.

The present study has some limitations. First, there is no adequate explanation for the decline in correlation between AHI and BEI in a more severe form of OSA. Second, the enrolled patient had increased risk of OSA, and thus, risk of SB was assessed in the cohort of sleep-disturbing breathing, not in general population. Third, the study group included a few patients with diabetes; hence, further studies in a group with more patients with diabetes are needed to confirm these new risk factor for SB.

## 5. Conclusions

The relationship between OSA and SB depends on the degree of severity of OSA. From the results of the present study, mild-to-moderate OSA is associated with SB in the group of patients with increased risk of OSA. Diabetes could be a new risk factor for SB.

## Figures and Tables

**Table 1:** Polysomnographic indices in the studied group (*n* = 110).

Parameter	Mean ± SD	Minimum	Maximum
SE (%)	80.48 ± 10.37	52.40	96.80
SL (min)	21.92 ± 20.70	0.00	112.60
WASO (min)	55.88 ± 38.96	3.00	172.50
N1 (% of TST)	5.93 ± 4.99	0.20	21.20
N2 (% of TST)	47.72 ± 9.79	26.20	72.90
N3 (% of TST)	24.61 ± 9.65	2.60	52.70
REM (% of TST)	21.75 ± 7.72	4.10	48.90
BEI (n/hour)	3.70 ± 4.27	0.0	24.70
Phasic BEI (n/hour)	1.93 ± 3.13	0.0	19.30
Tonic BEI (n/hour)	1.13 ± 1.30	0.0	6.90
Mixed BEI (n/hour)	0.66 ± 0.79	0.0	4.00
AHI (n/hour)	23.28 ± 19.98	0.0	100.10
ODI (n/hour)	22.92 ± 19.60	0.0	83.40
Mean SatO_2_ (%)	92.68 ± 2.19	83.30	96.80
Minimal SatO_2_(%)	81.76± 7.39	54.00	93.00
Cheyne-Stokes (% of TST)	0.69 ± 2.41	0.0	18.20
Mean desaturation (%)	4.78± 2.28	3.0	19.80

SE: sleep efficiency, SL: sleep latency, WASO: wake after sleep onset, REM: rapid eye movement, BEI: bruxism episode index, AHI: apnea–hypopnea index, ODI: oxygen desaturation index, SatO_2_: oxygen saturation.

**Table 2:** The prevalence of obstructive sleep apnea and sleep bruxism in the studied group.

Parameter	%	*n*	
AHI (n/hour)	<5	13.63	15
	≥5<15	30.0	33
	≥15<30	25.44	28
	≥30	30.90	34
BEI (n/hour)	<2	50	55
	≥2<4	20	22
	≥4	30	33

BEI: bruxism episode index, AHI: apnea–hypopnea index.

**Table 3:** The correlations between polysomnographic indices and BEI in the group with mild and moderate OSA (AHI < 30).

Parameter	BEI (n/hour)	Phasic BEI (n/hour)	Tonic BEI (n/hour)	Mixed BEI (n/hour)
AHI (n/hour)	**0.24**	**0.27**	0.03	0.15
SL (min)	−0.11	−0.08	−0.07	−0.16
WASO (min)	−0.06	−0.01	−0.15	−0.08
SE (%)	0.17	0.16	0.14	0.07
N1 (% of TST)	0.10	0.10	−0.07	0.19
N2 (% of TST)	−0.01	−0.01	−0.00	−0.01
N3 (% of TST)	0.08	0.06	0.10	0.05
REM (% of TST)	−0.15	−0.12	−0.09	−0.17
Arousal index (n/hour)	**0.45**	**0.52**	−0.08	**0.34**
Cheyne-Stokes (% of TST)	0.09	0.07	−0.13	−0.05
ODI (n/hour)	0.20	**0.23**	0.02	0.14
SatO_2_ (%)	−0.02	−0.06	0.09	0.02
Min SatO_2_ (%)	−0.20	**−0.26**	0.08	−0.11
Mean desaturation (%)	−0.04	0.03	−0.07	0.0

SE: sleep efficiency, SL: sleep latency, WASO: wake after sleep onset, REM: rapid eye movement, BEI: bruxism episode index, AHI: apnea–hypopnea index, ODI: oxygen desaturation index, SatO_2_: oxygen saturation; statistically significant differences are marked as bold (*p* < 0.05).

**Table 4:** The results of estimation for the models obtained with multivariate regression analysis in group with AHI < 30.

Parameter	Models for BEI			
		Model 1		
	Rc	SEM of RC	*p*	R^2^
intercept	**0.92**	**0.69**	**0.024**	**0.488**
AHI	**0.12**	**0.03**	**0.029**	
Arousal index	0.23	0.17	0.076	
Male gender	**1.73**	**0.72**	**0.034**	
Age	0.03	0.03	0.115	
Diabetes	**1.59**	**1.21**	**0.039**	
Coronary artery disease	1.25	1.66	0.201	
		Model 2		
	Rc	SEM of RC	*p*	R^2^
intercept	**1.28**	**0.68**	**0.013**	**0.471**
AHI	**0.09**	**0.03**	**0.030**	
Arousal index	0.24	0.15	0.071	
Male gender	**1.80**	**0.82**	**0.045**	
Age	0.04	0.03	0.285	
Diabetes	**1.43**	**1.07**	**0.036**	
		Model 3		
	Rc	SEM of RC	*p*	R^2^
intercept	**1.65**	**1.03**	**0.007**	**0.458**
AHI	**0.08**	**0.03**	**0.034**	
Arousal index	0.25	0.17	0.070	
Male gender	**1.97**	**0.91**	**0.035**	
Diabetes	**1.47**	**0.94**	**0.040**	
		Model 4		
	Rc	SEM of RC	*p*	R^2^
intercept	**1.83**	**1.06**	**0.004**	**0.423**
AHI	**0.10**	**0.04**	**0.044**	
Male gender	**2.22**	**0.96**	**0.024**	
Diabetes	**1.47**	**1.06**	**0.047**	

AHI: apnea–hypopnea index, Rc: regression coefficient, SEM: standard error of mean; statistically significant differences are marked as bold (*p* < 0.05).

## References

[1] Hou H., Zhao Y., Yu W., Dong H., Xue X., Ding J., Xing W., Wang W. (2018). Association of obstructive sleep apnea with hypertension: A systematic review and meta-analysis. J. Glob. Health.

[2] Munoz R., Duran-Cantolla J., Martinez-Vila E., Gallego J., Rubio R., Aizpuru F., De La Torre G. (2006). Severe sleep apnea and risk of ischemic stroke in the elderly. Stroke.

[3] Vasheghani-Farahani A., Kazemnejad F., Sadeghniiat-Haghighi K., Saadat S., Tavakolipoor P., Yazdani T., Alidoosti M., Ghasem-Amooeian V., Ashaf H. (2018). Obstructive sleep apnea and severity of coronary artery disease. Caspian J. Intern. Med..

[4] Mehra R., Benjamin E.J., Shahar E., Gottlieb D.J., Nawabit R., Kirchner H.l., Sahadevan J., Redline S. (2006). Association of nocturnal arrhythmias with sleep-disordered breathing: The Sleep Heart Health Study. Am. J. Respir. Crit. Care. Med..

[5] Young T., Finn L., Peppard P.E. (2008). Sleep disordered breathing and mortality: Eighteen-year follow-up of the Wisconsin sleep cohort. Sleep.

[6] Hollowell D.E., Bhandary P.R., Funsten A.W., Suratt P.M. (1991). Respiratory related recruitment of the masseter: Response to hypercapnia and loading. J. Appl. Physiol..

[7] Senaratna C.V., Perret J.L., Lodge C.J., Lowe A.J., Campbell B.E., Matheson M.C., Hamilton G.S., Dharmage S.C. (2017). Prevalence of obstructive sleep apnea in the general population: A systematic review. Sleep Med. Rev..

[8] Lobbezoo F., Ahlberg J., Raphael K.G., Wetselaar P., Glaros A.G., Kato T., Santiago V., Winocur E., De Laat A., De Leeuw R. (2018). International consensus on the assessment of bruxism: Report of a work in progress. J. Oral Rehabil..

[9] American Academy of Sleep Medicine (2014). International Classification of Sleep Disorders.

[10] Sateia M.J. (2014). International classification of sleep disorders-third edition: Highlights and modifications. Chest.

[11] Klasser G.D., Rei N., Lavigne G.J. (2015). Sleep bruxism etiology: The evolution of a changing paradigm. J. Can. Dent. Assoc..

[12] Lavigne G., Manzini C., Huynh N.T., Kryger M.H., Roth T., Dement W.C. (2011). Sleep bruxism. Principles and Practice of Sleep Medicine.

[13] Kuhn M., Türp J.C. (2018). Risk factors for bruxism. Swiss. Dent. J..

[14] Hosoya H., Kitaura H., Hashimoto T., Ito M., Kinbara M., Deguchi T., Irokawa T., Ohisa N., Ogawa H., Takano-Yamamoto T. (2014). Relationship between sleep bruxism and sleep respiratory events in patients with obstructive sleep apnea syndrome. Sleep Breath..

[15] Jokubauskas L., Baltrušaitytė A. (2017). Relationship between obstructive sleep apnoea syndrome and sleep bruxism: A systematic review. J. Oral Rehabil..

[16] Saito M., Yamaguchi T., Mikami S., Watanabe K., Gotouda A., Okada K., Hishikawa R., Shibuya E., Shibuya Y., Lavigne G. (2016). Weak association between sleep bruxism and obstructive sleep apnea. A sleep laboratory study. Sleep Breath..

[17] Sjöholm T.T., Lowe A.A., Miyamoto K., Fleetham J.A., Ryan C.F. (2000). Sleep bruxism in patients with sleep-disordered breathing. Arch. Oral Biol..

[18] Berry R.B., Budhiraja R., Gottlieb D.J., Gozal D., Iber C., Kapur V.K., Marcus C.L., Mehra R., Parthasarathy S., Quan S.F. (2012). American Academy of Sleep Medicine. Rules for scoring respiratory events in sleep: Update of the 2007 AASM Manual for the Scoring of Sleep and Associated Events. Deliberations of the Sleep Apnea Definitions Task Force of the American Academy of Sleep Medicine. J. Clin. Sleep Med..

[19] Lavigne G.J., Rompre P.H., Montplaisir J.Y. (1996). Sleep bruxism: Validity of clinical research diagnostic criteria in a controlled polysomnographic study. J. Dent. Res..

[20] Sousa H.C.S., Lima M.D.M., Dantas N.N.B., Tobias R.Q., Moura M.S., Moura L.F.A.D. (2018). Prevalence and associated factors to sleep bruxism in adolescents from Teresina, Piauí. Rev. Bras. Epidemiol..

[21] Manfredini D., Guarda-Nardini L., Marchese-Ragona R., Lobbezoo F. (2015). Theories on possible temporal relationships between sleep bruxism and obstructive sleep apnea events. An expert opinion. Sleep Breath..

[22] Ohayon M.M., Li K.K., Guilleminault C. (2001). Risk factors for sleep bruxism in the general population. Chest.

[23] Kato T., Velly A.M., Nakane T., Masuda Y., Maki S. (2012). Age is associated with self-reported sleep bruxism, independently of tooth loss. Sleep Breath..

[24] Gold A.R., Dipalo F., Gold M.S., O’Hearn D. (2003). The symptoms and signs of upper airway resistance syndrome: A link to the functional somatic syndromes. Chest.

[25] Bader G., Lavigne G. (2000). Sleep bruxism; an overview of an oromandibular sleep movement disorder. Sleep Med. Rev..

[26] Tan M.W.Y., Yap A.U., Chua A.P., Wong J.C.M., Parot M.V.J., Tan K.B.C. (2018). Prevalence of Sleep Bruxism and Its Association with Obstructive Sleep Apnea in Adult Patients: A Retrospective Polysomnographic Investigation. J. Oral Facial Pain Headache.

[27] Mengatto C.M., Dalberto Cda S., Scheeren B., Barros S.G. (2013). Association between sleep bruxism and gastroesophageal reflux disease. J. Prosthet. Dent..

[28] Nakata A., Takahashi M., Ikeda T., Hojou M., Araki S. (2008). Perceived psychosocial job stress and sleep bruxism among male and female workers. Community Dent. Oral Epidemiol..

[29] Bisulli F., Vignatelli L., Naldi I., Licchetta L., Provini F., Plazzi G., Di Vito L., Ferioli S., Montagna P., Tinuper P. (2010). Increased frequency of arousal parasomnias in families with nocturnal frontal lobe epilepsy: A common mechanism?. Epilepsia.

[30] Winocur E., Uziel N., Lisha T., Goldsmith C., Eli I. (2011). Self-reported bruxism—Associations with perceived stress, motivation for control, dental anxiety and gagging. J. Oral Rehabil..

[31] Agashe S., Petak S. (2018). Cardiac Autonomic Neuropathy in Diabetes Mellitus. Methodist Debakey Cardiovasc. J..

[32] López-Pintor R.M., Casañas E., González-Serrano J., Serrano J., Ramírez L., de Arriba L., Hernández G. (2016). Xerostomia, Hyposalivation, and Salivary Flow in Diabetes Patients. J. Diabetes Res..

[33] Dumais I.E., Lavigne G.J., Carram M.C., Rompré P.H., Huynh N.T. (2015). Could transient hypoxia be associated with rhythmic masticatory muscle activity in sleep bruxism in the absence of sleep-disordered breathing? A preliminary report. J. Oral Rehabil..

[34] Manfredini D., Serra-Negra J., Carboncini F., Lobbezoo F. (2017). Current concepts of bruxism. Int. J. Prosthodont..

